# Comprehensive rehabilitation and job reintegration of people with severe mental illness in a Latin American country: REINTEGRA study protocol

**DOI:** 10.1186/s12888-023-04835-4

**Published:** 2023-06-19

**Authors:** Jaime Carmona-Huerta, Sol Durand-Arias, Elsy Cárdenas-García, J. Carlos Arámbula-Román, Marianne Guzmán-Ramírez, Isabel Estrada-Ramírez, María Trinidad Amezcua-Ramírez, Verónica Lastra-González, Santiago Castiello-de Obeso, Alejandro Aldana-López

**Affiliations:** 1 Jalisco Institute of Mental Health, SALME, Guadalajara, Mexico; 2 University of Guadalajara, University Center of Health Sciences, Guadalajara, Mexico; 3 National Institute of Psychiatry Ramón de La Fuente Muñiz, Ciudad de México, Mexico; 4 Benemérita Universidad Autónoma de Puebla, Puebla, Mexico; 5 Corpus Christi College, University of Oxford, Oxford, UK

**Keywords:** Severe mental illness, Psychosocial rehabilitation, Job reinsertion, Comprehensive rehabilitation

## Abstract

**Background:**

Despite the increasing use of comprehensive rehabilitation models for people with severe mental illness (SMI), there are still limitations to their implementation and replicability in a consensual way, particularly in Latin American countries. The REINTEGRA program aims to be a standardized model of comprehensive rehabilitation focused on psychosocial and cognitive improvement through a set of interventions on different areas of people’s functionality, with the goal of reintegrating people with SMI into the labour market. In this paper we summarize the protocol for its subsequent implementation in a mental health institution in Mexico.

**Method:**

The protocol is based on a quasi-experimental, prospective longitudinal study, with a pragmatic or naturalistic control group. It will be carried out in three phases. Phase 1 consists of a series of interventions focused on psychosocial improvement; Phase 2 focuses on cognitive and behavioral improvement treatments; and Phase 3 targets psychosocial recovery through rehabilitation and reintegration into the labour market. The overall procedure will be monitored with standarized evaluations at different stages of the program.

**Discussion:**

This study presents a model of integral rehabilitation of people with SMI. At the moment, one of the obstacles to overcome is the organization and procedural control of the different actors needed for its implementation (nurses, psychologists, doctors, companies, institutions, etc.). REINTEGRA will be the first comprehensive rehabilitation model that includes systematized procedures for job reinsertion for people with SMI in Mexico, which aims to be a standardized tool of easy adaptation and the replicability for other mental health centers and institutions.

## Background

According to the Substance Abuse and Mental Health Services Administration (SAMHSA) [1], severe mental illnesses (SMI) are defined as the mental, behavioral, or emotional disorders (excluding neurodevelopmental and substance use disorders) that lead to a serious impairment of functioning, i. e., that interfere with or limit one or more activities of daily living. SMI affect at least 2% of the world’s population, and one of their main consequences—in the medium and long term—is the loss of global functionality, represented by difficulties in social interaction, cognitive impairment, and behavioral errors, as well as segregation, stigma, and loss of job opportunities.

Increasingly, global models of mental health promotion and intervention consider not only individual factors (psychological and biological), but also structural factors and social development. The World Health Organization (WHO) [2] mentions that, to act on the determinants of mental health, it is necessary to adopt measures that involve the sectors responsible for education, labor, justice, transportation, etc., focusing on the individual and considering their life plan and environment, by promoting and strengthening their autonomy and independence. Thus, the recovery of individuals implies changing the catastrophic vision of mental illness to achieve functional development, identifying the aspirations and personal goals of those suffering from some type of SMI. In this sense, community rehabilitation and reintegration are considered of vital importance, based on individual needs for the development of skills and competencies that facilitate access to labor market, considering labor insertion as a precursor of social inclusion in people with SMI [3].

In order to make a complete and objetive psychosocial assessment of individuals, it is necessary to address two key concepts: functioning and disability. The International Classification of Functioning, Disability and Health (ICF), developed by WHO, defines functioning as a global term that refers to all bodily functions, activities, and social participation; disability on the other hand, includes deficiencies, limitations in activity or restrictions in participation. For this reason, it is important to note that a person’s degree of functioning and disability does not depend only on the presence or absence of physical or mental symptoms, but also on personal and environmental barriers or facilitators to a particular disease. Therefore, individuals who have a disease or condition that causes disability may benefit not only from interventions aimed at reducing symptoms, but also from interventions designed to prevent and/or modify functional impairment and contextual barriers [4, 5].

As an example of this, we can mention schizophrenia and bipolar disorder, which are disorders that alter affective, cognitive, and behavioral processes that affect both the individual and society and, therefore, can create disabilities of different kinds in people who suffer from it [6]. In fact, according to WHO, these disorders are among the conditions that lead to the most lost working days due to disability [7]. Consequently, the approach aimed at the recovery of people with this type of illness must consider the training and rehabilitation of the cognitive, behavioral, and social spheres, which are oriented towards independence and community reintegration [8].

In the case of Mexico, generally speaking, medical management is provided with favorable results for mental disorders; however, up to 75% of those who suffer from the do not receive the required psychosocial care, thus it is important to develop services focused on mental health at a community level, as well as to focus on the first level in order to reduce the diagnostic and treatment gap [9].

Regarding SMIs, their chronic nature represents high costs for the health system, to which are added high levels of disability, family strain and social dysfunction. In addition, a pattern of marginalization, isolation and exclusion from the labor market has been observed among people with SMI who suffer from cognitive impairment. The foregoing highlights the need to make a change in the model of care, in such a way that it focuses on the person and their needs, that it is provided within their community and that a special emphasis is placed on functional reintegration, social, cognitive, behavioral, and occupational rehabilitations [10–12].

Although there are different models with the vision of integral rehabilitation in different parts of the world [3, 13], there is not enough evidence for its use by consensus, or its level of replicability is low, given that there are no manuals for its implementation, and there are no adaptations for Latin American countries. In the case of Mexico, attention to these disorders has had a deficient approach, with and unconnected mental health system focused solely on crisis management and pharmacological treatment of the symptoms of the pathology in psychiatric hospitals. However, as mentioned, there is no published, validated, manualized, replicated or evidence-based rehabilitation program that, in a standardized and consensus-based way by experts, develops an action protocol that brings together in an articulated way the different multidisciplinary actors (psychologists, psychiatrists, nurses, social workers, etc.) through a comprehensive management aimed at the occupational reintegration and functional recovery of people with SMI [9].

Given the above, the aim of this paper is to present the REINTEGRA protocol for the standardization and implementation of the rehabilitation and labor reintegration for people with SMI, which is based on the articulation, systematization and implementation of various training-related interventions in the various functional areas of individuals, and whose effectiveness has been previously demonstrated: psychoeducation, training in activities of daily living (ADL), social skills training (SST), computer-based cognitive remediation (CBCR), metacognitive therapy (MCT), recovery-oriented cognitive behavioural therapy (CT-R) and skills oriented to job integration (SOJI). They are briefly described below.

- Psychoeducation: Developed by Anderson in 1980; it is the process by which health professionals collaborate with service users and their family members for the latter to acquire knowledge about the disease and skills to maintain the best possible level of mental health [14].
- Training in ADL: According to Reed and Sanderson [15], ADL is defined as “the tasks that a person must be able to perform to take care of himself, including self-care, communication, and movement” [16].
- SST: Refers to behaviors that, combined in an appropriate sequence and used in appropriate ways and places, enable the individual to be successful in social skills [16, 17].
- CBCR: Its aim is to reduce the cognitive impairment that can cause various disorders, favoring neuronal plasticity, enhancing preserved functions, and recovering those that are not preserved [14, 17].
- MCT: It is focused on improving metacognition, which is defined as the active examination and reflection of the cognitive processes themselves, the observation of their products and the study of the reasoning generated [18, 19].
- CT-R: It is an adaptation of Beck’s cognitive behavioral therapy focused on the recovery of people suffering from schizophrenia with predominantly negative symptoms and low social functioning [20].
- SOJI: Based on the individual objectives of each participant, this training uses modeling techniques, workshop activities and links to job reintegration programs.

Based on these premises and on the understanding that people with SMI have low levels of autonomy, poor interpersonal relationships, deficient management of leisure time, difficulty in obtaining and maintaining a job, and complications in administering their finances, reintegration into the labor market is considered one of the main outcomes of REINTEGRA. The difficulties faced by people with these illnesses are similar throughout the world; their reintegration and occupational prognosis are directly related to symptomatological stability, therapeutic adherence, positive attitude, and family support. Greater social awareness and corporate awareness are needed to prevent discrimination. In the workplace, it is necessary to adapt working hours to flexible hours and reduce the stress burden [21].

## Method

### Objective

To evaluate the effectiveness of the implementation of the REINTEGRA program, which includes cognitive, behavioral, social, and occupational rehabilitation of people with SMI.

### Design

**Fig. 1:**
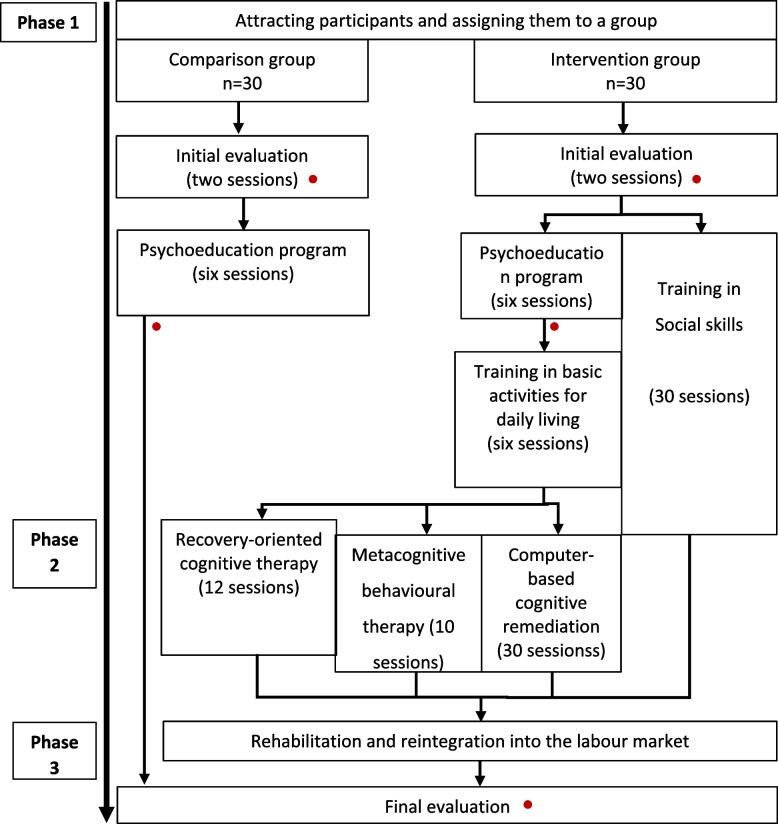
Test Desing of the REINTEGRA project

Quasi-experimental prospective, longitudinal study (with control group without random assignment), of pragmatic or naturalistic type. The REINTEGRA program was designed with the intention of intervening in the participants’ usual environment to give greater weight to external validity, since the aim is to transfer it into daily practice with the expectation of obtaining the same effectiveness as in the original design. Figure 1 summarizes the process that REINTEGRA participants will follow. The program is designed to be developed in three phases: Phase 1 will carry out initial assessments, psychoeducation program, SST, and training in ADL; Phase 2 will include the application of CBCR, MCT and CT-R programs; Phase 3 will include the application of SOJI training and the process of job insertion.

Both the informed consent and the present study will be carried out in accordance with the scientific and ethical principles of beneficence, non-maleficence, autonomy and justice for research in humans in accordance with the Declaration of Helsinki and its amendments, having been approved by the Research and Ethics Committee of the Mental Health Institute of Jalisco (SALME), number: 205–2021.

### Scenery

All phases of REINTEGRA will be carried out at Centro de Atención Integral de Salud Mental Estancia Prolongada, by its acronym in Spanish (CAISAME-EP), which is part of the Jalisco Mental Health Institute (SALME). The multidisciplinary team of CAISAME-EP will include psychiatrists, general practitioners, psychologists, nurses, and social workers, who will be trained in the same facilities of CAISAME-EP for the proper implementation of the program.

### Participants

Men and women aged 18 to 55 who have been diagnosed with SMI and are being treated at SALME will be offered to participate in the study, whether they are hospitalized and about to be discharged or receiving outpatient care but who, at the discretion of the attending physician, are candidates for rehabilitation programs. Interventions and sequential steps of the protocol are described in the Assesment tools and Procedures section.

The sample size was calculated using the formula for contrasting hypotheses on two proportions [22], where the expected success rate is 40% (p1 = 0.40) for the intervention group, compared to a rate of 10% (p2 = 0.10) reported under normal treatment conditions. A significance level of 5% (α = 0.05) and a statistical power of 80% (β = 0.80) were considered. As a result, a sample of 19 subjects per group was obtained, but due to the expected losses and the need to perform parametric tests, it was decided to increase the sample number to 30 subjects per group.

$${n}_{0 }=\frac{{\left({Z}_{\alpha }\sqrt{2p\left(1-p\right)}+ {Z}_{\beta }\sqrt{{p}_{1}\left(1- {p}_{1}\right)+ {p}_{2}\left(1- {p}_{2}\right)}\right)}^{2}}{{({p}_{1}- {p}_{2})}^{2}}$$

### Inclusion criteria

1) SALME users diagnosed with SMI (schizophrenia, bipolar disorder, schizoaffective disorder and other non-organic psychoses) with at least two years of history, according to the International Classification of Diseases (ICD-10); 2) that they do not carry out any remunerated activity; 3) under pharmacological treatment and control of symptoms; 4) achieving a score > 50 on the Global Assessment of Functioning (GAF, integrated into the DSM-IV-TR); 5) who can read and write; 6) commitment to attend the sessions required by REINTEGRA (only for the intervention group).

### Exclusion criteria

1) Active use of non-pharmacological psychoactive substances other than tobacco, 2) diagnosis of intellectual development disorders and/or moderate or severe cognitive impairment, 3) suffering from chronic diseases that limit work capacity (cerebrovascular disease, renal failure, etc.), 4) presenting disruptive behaviour and/or active suicidal behaviour.

### Elimination criteria

1) Withdrawal of informed consent, 2) worsening of the disorder that needs hospitalization, 3) incomplete initial assessment, 4) failure to meet at least 75% of the attendance at the REINTEGRA program, 5) development of a medical condition that limits their stay in the program.

### Assessment tools

At the beginning of the program, the sociodemographic data card will be completed, which includes age, sex, education, marital status, and religion; the Clinical Global Impression of schizophrenia (CGI-SCH) scale that quickly and easily measures positive, negative, depressive, and cognitive symptoms of schizophrenia will be applied monthly.

The other instruments will be applied at the beginning, after each phase and at the end of the program, according to their relevance (Table 1): the clinical data card (therapeutic adherence, clinical incidents per week, discharge from the intervention, relapse or rehospitalization, etc.); the Brief Psychiatric Rating Scale (BPRS), efficiently evaluates changes in symptoms such as somatic worries, psychological anxiety, emotional isolation, conceptual disorganization, among others; The Young Mania Rating Scale (YMRS), helps monitor euphoria, hyperactivity, sleep, irritability, among other symptoms during the last 48 h; Patient Health Questionnaire (Depression Scale, PHQ-9), is self-applicable and consists of nine items that assess the presence of depressive symptoms. The Social Occupational Functioning Scale (SOFS), which assesses self-care and activities of daily living, communication, interpersonal relationships and instrumental skills for life and work; the Internalized Stigma of Mental Illness (ISMI) scale, which evaluates stigma through three subscales: discrimination, revelation and positive aspects of mental illness; the Level of express emotion (LEE) is self-applicable and is directed to the patient’s family in order to obtain information about the family environment; the Recovery Assessment Scale (RAS 24) aims to assess the patient’s recovery expectations; WHO Quality of Life Survey (WHOQOL-BREF), provides a quality of life profile considering physical, psychological, social and environmental aspects. And finally, the Consensus Cognitive Battery MATRICS (MCCB MATRICS), which includes ten optimal tests to evaluate cognitive domains such as: processing speed, verbal fluency, attention, working memory, verbal and visual memory, reasoning and problem solving, among others. All instruments have their versions translated into Spanish and validated for the population of interest [23–32].

**Table 1:** List of application of the scales for each of the phases of the project

SCALE	**Comparison group**			**Intervention group**					
	PHASE 1		PHASE 3	PHASE 1			PHASE 2		PHASE 3
	At the beginning of the IE	At the end of the PE program	At the end	At the beginning of the IE	At the end of the PE program	At the end of ADL training	At the end of CT-R	At the end	At the end
Socio-demographic data card	**x**			**x**					
Clinical data card ^a^	**x**	**x**	**x**	**x**	**x**	**X**	**x**	**x**	**x**
BPRS	**x**		**x**	**x**					**x**
CGI-SCH^a^	**x**	**x**	**x**	**x**	**x**	**X**	**x**	**x**	**x**
YMRS	**x**		**x**	**x**		**X**	**x**	**x**	**x**
PHQ-9	**x**		**x**	**x**		**X**	**x**	**x**	**x**
SOFS	**x**	**x**	**x**	**x**	**x**	**X**	**x**	**x**	**x**
ISMI	**x**	**x**	**x**	**x**	**x**	**X**	**x**	**x**	**x**
LEE	**x**	**x**	**x**	**x**	**x**				**x**
RAS	**x**	**x**	**x**	**x**	**x**	**x**	**x**	**x**	**X**
WHOQOL-BREF	**x**	**x**	**x**	**x**	**x**	**x**	**x**	**x**	**X**
MATRICS	**x**		**x**	**x**					**X**

*IE* Initial evaluation, *PE* Psychoeducational (see the rest of the abbreviations in the corresponding section at the end of the article)

Scales with ^a^ shall be applied each month

### Procedure

The program proposed contemplates a standardized structure in three phases:

### Phase 1

Attracting participants and assignment to intervention/comparison group. Once the attending physician identifies a potential participant, a member of the research team will explain what their participation in the program would be like and assess whether they adequately meet the inclusion and exclusion criteria. Participants who meet these criteria will be invited to participate in the intervention and will be divided into two groups: intervention (IG) and comparison (CG).

Those who meet the inclusion and exclusion criteria, and who also agree to participate in all phases of the program, will be part of the IG; the corresponding letter of informed consent will be obtained from the first 30 people who commit to adhering to the intervention. Those who do not agree to adhere to the intervention will be part of the CG, these participants will be given longitudinal follow-up, will have their usual management by their multidisciplinary mental health team and will be applied psychometric scales for comparison purposes, as well as a face-to-face psychoeducation course aimed at both the participant and their families (the same course will be applied to the IG). The decision not to carry out the random assignment is because participation in the intervention group requires the commitment of a strict adherence to the different interventions (compliance of at least 80%), so the IG would have a high risk of follow-up bias if the participants were not sure that they would be able to comply with it. Furthemore, forming a CG with participants who wanted to be part of the IG would mean denying them the possibility of receiving training that would be beneficial to them.

It should be noted that, regardless of the group to which they belong, participants should maintain their usual pharmacological treatment and be under control of the symptomatology of the disease. In addition, at the end of the study, comparison group members will be invited back to join the full program if they wish, thus resolving any ethical dilemmas.

### Initial assessment

Once the participant is assigned to a group, the initial assessment, which consists of two sessions, will begin. In the first, sociodemographic and clinical data will be obtained and the scales BPRS, YMRS, PH1-9, SOFS, LEE, RAS and WHOWQOL-BREF will be applied; in the second, the MATRICS neuropsychological battery will be administered and goals will be set (intervention in case of IG and usual treatment for CG), based on the clinical and job history of each person, and on their individual characteristics and needs, taking into account environmental factors that may influence their process.

### Psychoeducation program

This is the only part of the program that will be applied to both groups; it will consist of six sessions on mental health, mental disorders, and their treatment. Each session will last 1.30 h and will be given by the social work team to the individual with SMI and their family members. Once this part of the program has been completed, the appropriate scales will be applied to assess its effectiveness (see Table 1).

### Training in ADL

This part of the program will last six weeks and aims to empower the individual to be autonomous, safe, and effective in various activities of daily life (i. e. self-care, household skills, use of money and use of leisure time, to name a few), based on their current level of functioning.

#### SST

It will be carried out in parallel with the rest of the phases. There will be two group sessions (average of 10 participants) per week, of one hour each, for 15 weeks; and a basic scheme will be followed consisting of greeting, weekly homework review (if applicable), presentation of the skill or topic to be addressed, session specific instructions, application of techniques to train the skill (direct exposure, follow-up of instructions, role-playing, modeling, etc.), session reflections and discussion, feedback, conclusions, farewell and weekly homework (if deemed necessary).

##### Phase 2

#### CT-R

It will be applied in groups, during twelve sessions (one per week), in which the following topics will be developed: framing Interview, psychoeducation of negative symptoms, functional analysis of negative symptoms, abulia, anhedonia, asociality, cognitive symptoms and self-efficacy, programming of activities, automatic thoughts, management of unexpected situations, and closing and analysis of results.

### Cognitive rehabilitation

It consists of two different procedures: (1) CBCR and (2) MCT. There is still uncertainty regarding the effectiveness of computer-based CR for the treatment of chronic SMIs.

On the other hand, MCT consists of ten total modules that address problematic thinking styles and congnitive biases that are closely related to delusions: increased internal attributions, jumping to conclusions, bias towards disconfirmatory evidence of initial beliefs, inability to take perspective or empathize with others, overconfidence in memory errors, depressive cognitive patterns, self-esteem, and stigma [33].

### Phase 3

Rehabilitation and reintegration into the labor market. Based on the job objective set during the initial assessment, a job reintegration plan will be drawn up, which will include training for job skills through modeling techniques, direct training, and linkage with job reintegration programs. Each participant in the intervention group will be integrated into community work activities in accordance with the worker, employer and health professional handbook developed for this purpose. To facilitate this phase, it has the support of various institutions like the National Employment Service Jalisco Delegation. In addition, self-employment adapted to personal context and preferences will be an option.

Because within REINTEGRA, the participant is expected to return to their usual context after the interventions carried out, it is considered essential to follow-up six months after the program has concluded. This will be carried out by telephone with the support of the CAISAME-EP social work team.

### Stastical analysis

Descriptive, bivariate, and multivariate analyses of the groups will be performed, considering pairing by main confounding variables (age sex, disease severity), as well as a stratified analysis by sex and socio-occupational background. The possible change in the clinical stability of the disorder, levels of functionality, internalized stigma, permanence of employment and all measures obtained through the scales will be analyzed.

Bayesian statistics shall be used for inferences regarding outcomes. Inferences will be carried out using hierarchical models since the same participant will have several measurements over time. The models will be adjusted using Stan within the rstan package in the R and SPSS statistical language. It was decided to use these statistical methods and present a 95% plausible interval, since this will allow us to accept the hypothesis as to whether outcomes differ between groups [34].

## Discussion

REINTEGRA is based on the articulation of a set of psychosocial and cognitive interventions that have been shown to be effective. In addition, these interventions were chosen because they are congruent with each other regarding the approach for people with SMI and have been ordered by phase depending on the type of functional area to which they are focused. On the other hand, since REINTEGRA is a systematized model, whose effectiveness will be evaluated through experimental control, it is expected that its replicability and adaptation will be feasible in other institutional contexts. However, one of the biggest challenges is the organizational work of the operational team (e. g. nurses, psychologists, doctors, etc.), as well as the creation of inter-institutional links to establish agreements on access to work for people with SMI. In this project, these obstacles have been considered and have been worked on at an earlier stage (Phase 0), which is recommended in case of future implementation in other contexts and will be described in detail in the procedures of the manuals. Finally, it is hoped that REINTEGRA will not only facilitate the reintegration into job and improve the quality of life of people with SMI, but also make these conditions visible in favor of a social vision without stigma.

## Data Availability

Not applicable.

## Abbreviations

ADL: Activities of daily living
CAISAME-EP: Centro de Atención Integral de Salud Mental Estancia Prolongada
BPRS: Brief Psychiatric Rating Scale
CGI: Clinical Global Impression-Schizophrenia
ICD: International Classification of Diseases
ICF: International Classification of Functioning, Disability and Health
LEE: Level of express emotion
ISMI: Internalized Stigma of Mental Illness
GAF: Global Assessment of Functioning
CG: Comparison group
IG: Intervention group
SOJI: Skills oriented to job integration
SST: Social Skills Training
MCCB: Consensus Cognitive Battery MATRICS
WHO: World Health Organization
PHQ-9: Patient Health Questionnaire
RAS: Recovery Assessment Scale
CBCR: Computer-based cognitive remediation
SAMHSA: Substance Abuse and Mental Health Services Administration
SALME: Jalisco Mental Health Institute
SOFS: Social Occupational Functioning Scale
CT-R: Recovery-oriented Cognitive Therapy
MCT: Metacognitive therapy
SMI: Severe Mental Illness
WHOQOL-BREF: WHO Quality of Life Survey
YMRS: Young Mania Rating Scale
